# Germline Mutations in *Mtap* Cooperate with *Myc* to Accelerate Tumorigenesis in Mice

**DOI:** 10.1371/journal.pone.0067635

**Published:** 2013-06-26

**Authors:** Yuwaraj Kadariya, Baiqing Tang, Liqun Wang, Tahseen Al-Saleem, Kyoko Hayakawa, Michael J. Slifker, Warren D. Kruger

**Affiliations:** 1 Cancer Biology Program, Fox Chase Cancer Center, Philadelphia, Pennsylvania, Unites States of America; 2 Immune Cell Development and Host Defense Program, Fox Chase Cancer Center, Philadelphia, Pennsylvania, Unites States of America; Sapporo Medical University, Japan

## Abstract

**Objective:**

The gene encoding the methionine salvage pathway methylthioadenosine phosphorylase (*MTAP*) is a tumor suppressor gene that is frequently inactivated in a wide variety of human cancers. In this study, we have examined if heterozygosity for a null mutation in *Mtap (Mtap^lacZ^)* could accelerate tumorigenesis development in two different mouse cancer models, *Eμ-myc* transgenic and *Pten^+/−^*.

**Methods:**

*Mtap Eμ-myc* and *Mtap Pten* mice were generated and tumor-free survival was monitored over time. Tumors were also examined for a variety of histological and protein markers. In addition, microarray analysis was performed on the livers of *Mtap^lacZ/+^* and Mtap*^+/+^* mice.

**Results:**

Survival in both models was significantly decreased in *Mtap^lacZ/+^* compared to *Mtap^+/+^* mice. In *Eµ-myc* mice, *Mtap* mutations accelerated the formation of lymphomas from cells in the early pre-B stage, and these tumors tended to be of higher grade and have higher expression levels of ornithine decarboxylase compared to those observed in control *Eµ-myc Mtap^+/+^* mice. Surprisingly, examination of *Mtap* status in lymphomas in *Eµ-myc Mtap^lacZ/+^* and *Eµ-myc Mtap^+/+^* animals did not reveal significant differences in the frequency of loss of Mtap protein expression, despite having shorter latency times, suggesting that haploinsufficiency of *Mtap* may be playing a direct role in accelerating tumorigenesis. Consistent with this idea, microarray analysis on liver tissue from age and sex matched *Mtap^+/+^* and *Mtap^lacZ/+^* animals found 363 transcripts whose expression changed at least 1.5-fold (P<0.01). Functional categorization of these genes reveals enrichments in several pathways involved in growth control and cancer.

**Conclusion:**

Our findings show that germline inactivation of a single *Mtap* allele alters gene expression and enhances lymphomagenesis in *Eµ-myc* mice.

## Introduction

MTAP is a metabolic enzyme in the methionine salvage pathway that converts the polyamine synthesis byproduct 5′-dideoxy-5′-methylthioadenosine (MTA) into adenine and methylthioribose-1-phosphate and is expressed in all tissues throughout the body [1], [2]. Loss of MTAP expression is frequent in a large number of different human tumors including leukemias, lymphomas, mesothelioma, lung carcinoma, pancreatic carcinoma, squamous cell carcinoma, biliary tract cancer, glioblastoma, osteosarcoma, and neuroendocrine tumors [3]–[15]. Loss rates range from 14% to 100% depending on the tumor type and the method used to assess MTAP loss.

The *MTAP* gene is frequently inactivated in human tumors by large homozygous deletion of the 9p21 region where both the *MTAP* and the *CDKN2A/ARF* tumor suppressor genes are located [16]. In mice, similar deletions occur in the same gene cluster that is located on chromosome 4 [17]–[19]. Since these deletions generally inactivate *CDKN2A/ARF* as well as *MTAP*, it was initially hypothesized that loss of *MTAP* in tumors was simply due to it being a co-incident bystander. However, a variety of studies now indicate that *MTAP* is a tumor suppressor gene in its own right. Re-expression of MTAP protein in *MTAP*-deleted MCF-7 breast adenocarcinoma cells results in loss of anchorage independent growth *in vitro* and the ability to form tumors when injected into SCID mice [20]. In addition, expression of MTAP in an *MTAP*-deleted melanoma cell line or gastric cancer cell line results in reduced invasion and migration *in vitro* [21], [22]. Mice heterozygous for a germline mutation in the mouse *MTAP* gene (*Mtap)* die prematurely of T-cell lymphoma with a mean age of onset of about 18 months [23]. Finally, it was recently reported that humans with germline MTAP mutations have Diaphyseal medullary stenosis with malignant fibrous histiocytoma, an autosomal-dominant syndrome characterized by bone dysplasia, myopathy, and bone cancer [24]. Taken together, these observations suggest that *MTAP* functions as a tumor suppressor gene independent of *CDKN2A/ARF*.

A potential mechanism by which loss of *MTAP* enhances tumor formation involves the link between *MTAP* and polyamine metabolism. Polyamines are small aliphatic amines essential for cell growth and are elevated in tumors [25]. The rate-limiting enzyme in the production of polyamines is ODC, which catalyzes the conversion of ornithine to putrescine. Deletion of *MTAP* results in up-regulation of ODC in both yeast and human cells [20], [26], and over-expression of ODC is sufficient to transform fibroblast cells *in vitro* and cause increased frequency of skin tumors in a transgenic mouse model [27], [28]. More recently, Nilsson et al. demonstrated that ODC was over-expressed in the *Eµ-myc* transgenic mouse model of lymphoma and that this over-expression was important for lymphomagenesis [29]. Also, it has been shown that the ODC inhibitor DFMO increased tumor-free survival in TH-MYCN mice, which over express MYCN in neural lineages and develop neuroblastomas [30]. Taken together, these studies suggest that over-expression of ODC contributes to transformation by the *Myc* oncogene.

In the studies described here, we have crossed *Mtap^lacZ/+^* mice with both *Eµ-myc* mice and *Pten^+/−^* mice and have characterized the offspring for tumor formation. There were three distinct goals of these studies: 1) Strengthen the hypothesis that *Mtap*-loss plays a functional role in tumor formation; 2) Create a mouse model to study the effects of *Mtap*-loss on tumor formation that had a significantly shorter latency period than the original *Mtap^lacZ/+^* strain; and 3) Test the hypothesis that loss of *Mtap* might accelerate tumorigenesis in *Eµ-myc* mice by causing over expression of ODC.

## Materials and Methods

### Mouse Breeding and Survival Analysis

*Mtap* mice were created and genotyped as previously described [23]. *Eµ-myc* mice were obtained from the lab of Dr. John Cleveland (Scripps) and genotyped as described in [29]. *Pten* mice were provided by Dr. Antonio Di Cristofano (Albert Einstein University) and genotyped as described in [31]. All mice were in C57BL6 background.

For *Eµ-myc* animals, animals were monitored for survival and tumor formation daily by visual inspection and palpation. In these animals, tumor formation was obvious as indicated by swelling around the neck and associated lethargy. When tumor or distress was detected, the animals were euthanized and necropsied. *Pten^+/−^* animals were monitored in a similar manner, but sometimes the animals died spontaneously without tumors being detected. In cases where the deceased animal was relatively fresh, necropsies were performed to determine if tumor was present at time of death. In cases where a tumor could not be confirmed at the time of necropsy, animals were censored for the purposes of survival analysis.

### Ethics Statement

All animal protocols were approved by the Fox Chase Cancer Center IACUC (Protocol #05-06) and done in compliance with NIH guidelines. Animals were monitored daily for signs of distress and suffering. If distress or tumors were detected, animals were euthanized by overdose with isoflurane.

### Immunohistochemistry

Autopsied materials were fixed in buffered formalin, embedded in paraffin, and processed as previously described [23]. Rat antibodies directed against mouse CD45R/B220 (BD Biosciences) were used at a 1∶200 dilution. Rat antibodies against Ki67 (Dako) were used at a 1∶100 dilution. Rabbit ODC polyclonal serum was obtained from Dr. Lisa Chantz (Penn State University) and was used at a concentration of 0.5 ng/*µ*l. For each, visualization was achieved by incubation followed by incubating with either a goat anti-rabbit or anti-rat biotinylated secondary antibody followed by incubation with streptavidin peroxidase and 3,3′-Daminobenzidine (Sigma-Aldrich) substrate chromogen. Each slide was assessed blindly by a by a trained pathologist specializing in lymphoma (T.A-S) using a 1–6 grading system in which the percentage of cells containing the graded feature (Burkitt’s-like nuclei, Ki67, or ODC) was determined. A grade of 1 equals <10% of cells testing positive, 2 = 10–30%, 3 = 30–50%, 4 = 50–70%, 5 = 70–90%, and 6<90%.

### FACS Analysis

FACS analysis on spleen from euthanized animals was performed as previously described [32], [33]. Single cell suspensions were made from bone marrow, spleen, lymph node and thymus and stained with fluorochrome (FL, PE, APC, Cy7-PE) coupled monoclonal antibodies in various combinations; CD19 (1D3), CD45R/B220 (RA3-6B2), CD93/AA4 (AA4.1), IgM (331.12), IgD (11–26), CD21 (7G6), CD23 (B3B4), CD24 (30F1), CD3 (500A-A2), CD4 (GK1.5), CD8 (53-6), CD5 (53–7.3). Most reagents were made in the laboratory of Richard R. Hardy, except for FL-CD21 from BD Pharmingen, and FL-PNA (peanut agglutinin) from Vector Lab. Analysis was performed using a BD Biosciences LSR II/DiVa flow cytometer, equipped with three-laser excitation (405, 488, 630 nm).

### Quantitative RT-PCR Assay for TdT, Cµ, and Mtap

For *TdT* and *Cµ* analysis, total RNA was prepared by sorting 10^5^ cells into “Solution D,” followed by cDNA preparation as previously described [34]. Gene expression was quantified by real-time PCR, in duplicate, using an ABI7500 thermal cycler, and ABI software was used to determine relative gene expression levels, using *ß*-actin as an internal control.

### Mtap Quantification

Mtap protein levels were detected by Western blot analysis using a MTAP monoclonal antibody (Santa Cruz Biotechnology) at a 1/1000 dilution. Signal was visualized by SuperSignal West Pico Chemiluminescent kit (Pierce), and signal was quantified using Alpha Innotech image analyzer. All levels were normalized to an alpha-actin internal control. Mtap expression <20% that of control samples was scored as Mtap^−^.

### Microarray Experiments

Livers from 100-day-old male *Mtap^+/+^* and *Mtap^lacZ/+^* mice were excised and put into RNAlater (Ambion). Total RNA was isolated using TRIzol reagent (Invitrogen, Carlsbad, CA) and further purified by RNeasy kit (Qiagen, Valencia, CA) according to manufacturer’s instructions. RNA quality was evaluated by electrophoresis on Agilent Bioanalyzer (Agilent). Five µg of total RNA was first transcribed into cDNA using the Invitrogen’s Superscript system with an oligo (dT)24 primer containing a T7 RNA polymerase promoter sequence at its 5′ end. After double stranded cDNA synthesis using DNA polymerase I, Biotin-labeled cRNA was generated by *in vitro* transcription using a GeneChip IVT Labeling Kit (Affymetrix) according to manufacturer’s instructions and then purified using the GeneChip Cleanup Module. Label target was fragmented to a size of 35–200 bases by metal-induced hydrolysis prior to hybridization. Target hybridization was performed in an Affymetrix hybridization oven at 45°C for 16 hours using an Affymetrix GeneChip Mouse Genome 430A 2.0 Array. After hybridization arrays were washed using the Affymetrix fluidics station and stained with streptavidin Phycoerythrin according to the Affymetrix protocol. Four bacterial and phage cRNA controls (BioB, Bio C, Bio D and Cre) were included in the hybridization buffer to serve as internal control for hybridization efficiency. Washed arrays were scanned on an Affymetrix GeneChip Scanner 3000. Data was normalized using RMA as previously described [35]. Array data can be accessed in the GEO repository, GSE44539.

### Pathway Analysis

For pathway analysis, we selected a set of differentially regulated genes based on the criteria that they exhibited at least a 50% change in mRNA levels and had a p-value <0.01 (FDR <0.29). This list, containing 363 probes, was then analyzed using both Web Gestalt Gene Analysis Toolkit V2 [36], and the Ingenuity Pathways Analysis software (IPA, Ingenuity Systems, http://www.ingenuity.com). Both the Web Gestalt and IPA software maps the enriched genes on various canonical pathways and determines if the number of hits in each pathway exceeds those estimated by chance. The Web Gestalt software gives both an unadjusted and an adjusted P-value, where the adjusted P-value represents the false discovery rate. The IPA software ranks each pathway (called networks) by a score that is the negative log of the P-value. The IPA software does not determine a false discovery rate.

### Statistics

P-values for contingency tables (2×2) were all performed using Fischer’s exact test (2-sided). Survival curve comparisons were assessed using the log-rank test supplied in GraphPad Prism 4.0 (http://www.graphpad.com). For comparison of continuous variables, a Student’s t-test (2-sided) was used.

## Results

### Mtap^lacZ/+^ Decreases Survival in Eµ-myc and Pten^+/−^ Mice

**Figure 1:**
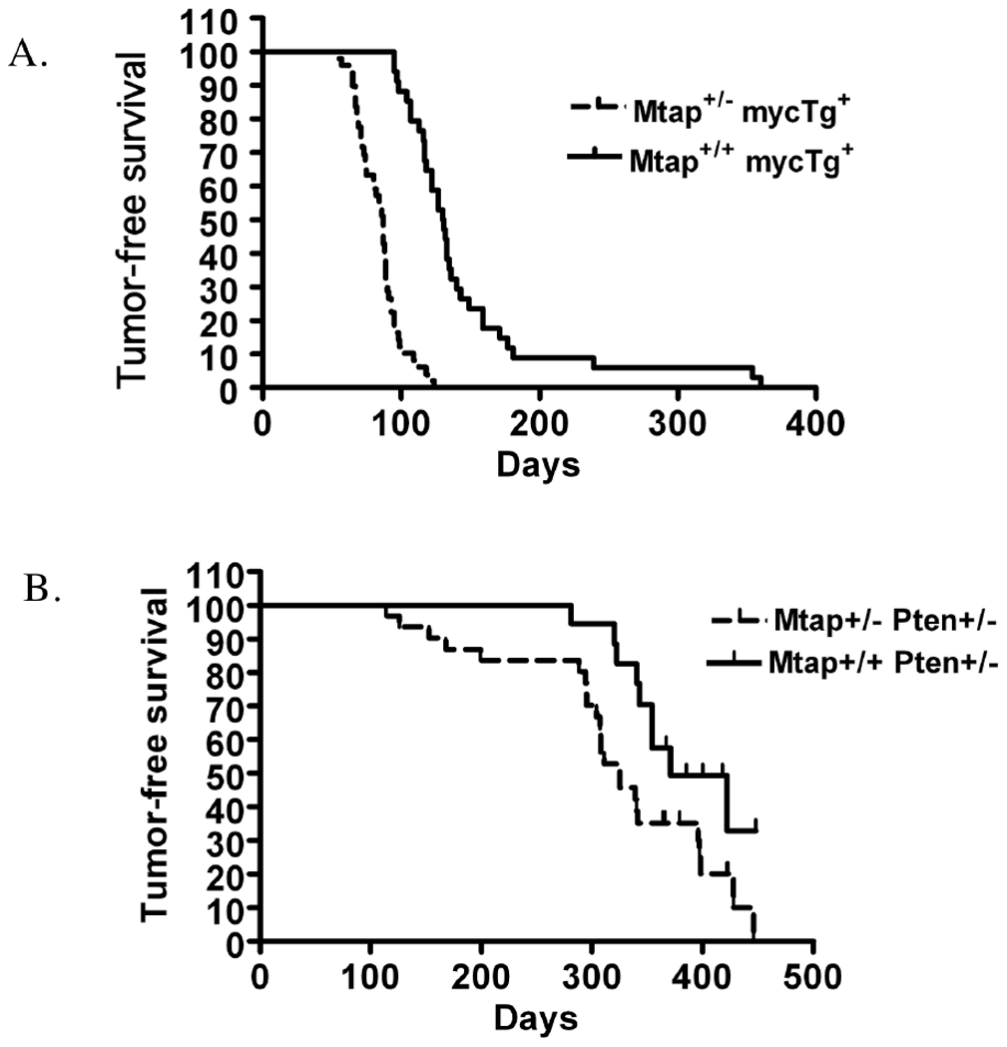
Mtap effect on tumor formation in *Eµ-Myc* and *Pten* ^+/−^ mice. A. Tumor-free survival of *Eµ-myc Mtap^+/+^* and *Eµ-myc Mtap^lacZ/+^* mice. Sibling *Eµ-myc Mtap^lacZ/+^* (n = 48) and *Eµ-myc Mtap^+/+^* (n = 42) were monitored daily for tumor formation and/or death for 368 days (p<0.001) B. Tumor-free survival of *Mtap^+/+^ Pten^+/−^* and *Mtap^lacZ/+^ Pten^+/−^* mice. Sibling of *Mtap^+/+^ Pten^+/+^* (n = 32) and *Mtap^lacZ/+^ Pten^+/−^* (n = 32) were monitored daily and followed for 450 days (p<0.031). Tick marks show censored observations (deaths with no detectable tumor).

Homozygous deletion of *Mtap* leads to early embryonic lethality [20]. However, *Mtap^lacZ/+^* mice are entirely normal until about 18 months of age when they will start to succumb to T-cell lymphoma. To address whether *Mtap* could accelerate tumorigenesis in other mouse tumor models, we crossed *Mtap^lacZ/+^* mice with *Eµ-myc* transgenic mice. *Eµ-myc* mice contain a transgene in which the immunoglobulin heavy chain enhancer is driving expression of the *Myc* oncogene in the B-lineage cells resulting in the development of B-cell lymphomas [37]. We followed a cohort *Eµ-myc Mtap^+/+^* and *Eµ-myc Mtap^lacZ/+^* littermates until they either developed visible lymphoma or died with disease as determined by necropsy. We found that *Eµ-myc Mtap^+/+^* animals had a median time to tumor formation of 130 days, compared to 87 days for *Eµ-myc Mtap^lacZ/+^* animals (P<0.001, Fig.1A). These results show that germline heterozygosity for *Mtap* significantly decreases tumor latency in *Eµ-myc* mice.

We also examined a second mouse tumor model, *Pten^+/−^*, for interaction with *Mtap*. *Pten* (Phosphatase and Tensin Homolog) is a phosphatase that dephosphorylates phosphatidylinositol (3,4,5)-trisphosphate resulting in the formation of phosphatidylinositol (4,5)-biphosphate, which causes inhibition of the AKT signaling pathway. Germline mutation in *PTEN* in humans is associated with Cowden’s disease, characterized by the presence of cutaneous benign hamartomas and high frequency of thyroid and breast cancer [38]. Homozygosity for *Pten* in mice is embryonic lethal, but heterozygous *Pten* mice (*Pten^+/−^*) develop a variety of tumors including lymphomas, endometrial tumors, colon cancer, and gonadal tumors that are detectable between six months and one year of age [31], [39], [40]. We crossed *Pten^+/−^* mice with *Mtap^lacZ/+^* animals and followed a cohort of *Mtap^+/+^ Pten^+/−^* and *Mtap^lacZ/+^ Pten^+/−^* for up to 450 days. We observed significantly decreased tumor-free survival in *Mtap^lacZ/+^* animals compared to *Mtap^+/+^* (median survival 325 vs. 371 days, P<0.031, Fig. 1B). We found there were significantly more spontaneous deaths in *Mtap^lacZ/+^* compared to *Mtap^+/+^* animals (Table 1). The most common type of tumor in both groups was lymphoma (10/32 and 4/32, respectively, Table 2). Other tumors observed included pheochromocytoma, thyroid, breast, and uterine adenocarcinomas. None of the individual tumors showed significant differences in occurrence between, between *Mtap^lacZ/+^* and Mtap^+/+^ animals, however, there was a significant difference between *Mtap^lacZ/+^* and *Mtap^+/+^* in the percentage of necropsied animals in which no lesion was detected (16% vs. 69%, P = 0.0001). These results show that heterozygosity for *Mtap* decreases survival in *Pten^+/−^* animals.

**Table 1:** Tumor formation and death in *Mtap Pten* animals.

Event	*Mtap^lacZ/+/^Pten^+/−^*	*Mtap^+/+^/Pten^+/−^*
Tumor formation determined by necropsy (median age)	16/32^a^	9/32^a^
	(50%)	(28.8%)
	(325 days)	(367 days)
Spontaneous death (autolysis)(median age) (median age)	11/32^b^	1/32^b^
	(34.37%)	(3.12%)
	(308 days)	(422 days)

a Mtap^lacZ/+^ vs. Mtap^+/+^, P = 0.125.

b Mtap^lacZ/+^ vs. Mtap^+/+^, P = 0.0027.

**Table 2:** Types of tumors in *Mtap^+/+^ Pten^+/−^* and *Mtap^lacZ/+^ Pten^+/−^* animals.

Tumor Type	*Mtap^lacZ/+^ Pten^+/−^*	*Mtap^+/+^ Pten^+/−^*
Lymphoma	10/32	4/32
Pheochromocytoma	3/32	2/32
Thyroid cancer	2/32	2/32
Breast cancer	0/32	1/32
Adenocarcinoma of uterus	1/32	0/32
No lesion detected	5/32	22/32^a^
Not necropsied	11/32	1/32^b^

a P<0.0001.

b P<0.0027.

### Mtap^lacZ/+^ Increases Grade, Proliferative Capacity, and Odc Expression in Eµ-myc Mice

**Figure 2:**
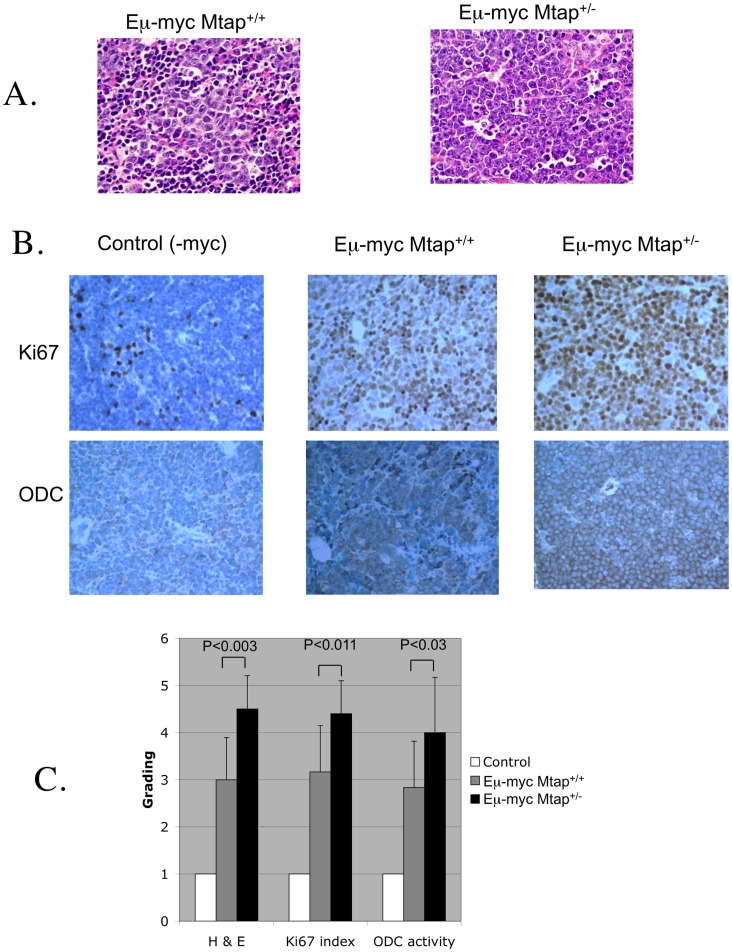
Pathology of *Eµ-myc Mtap* ^+/+^ and *Eµ-myc Mtap^lacZ/+^* mice. A. Representative H and E staining to tumor infiltrated thymus from *Eµ-myc Mtap^+/+^* and *Eµ-myc Mtap^lacZ/+^* animals viewed under 400X magnification. B. Representative Ki67 and ODC staining from the thymus of control, *Eµ-myc Mtap^+/+^* and *Eµ-myc Mtap^lacZ/+^* mice. C. Histologic grading from H and E, Ki67, and ODC. Grading was performed blinded and evaluated by a board certified clinical histopathologist specializing in hematological tumors (AS). A score of 1 is normal, while a score of 5 was the most severe. Error bars show SD of score for each group.

We next characterized the pathology of the lymphomas in *Eµ-myc* mice. First, we examined thymus sections from control, *Eµ-myc Mtap^+/+^,* and *Eµ-myc Mtap^lacZ/+^* animals for a variety of morphological and immunohistochemical features. As expected, staining with anti-bodies to either CD3 (T-cell marker) or CD45R/B220 (B-cell marker) indicated that all the lymphomas in both *Eµ-myc Mtap^+/+^* and *Eµ-myc Mtap^ +/−^* animals were B-cell neoplasms (not shown). Morphologically, these lymphomas exhibited a spectrum between large cells, with irregular nuclear membranes, vesicular chromatin, and prominent nucleoli (diffuse large B-cell lymphoma-like) to others with medium sized cells, relatively fine chromatin and small nucleoli with brisk mitotic activity and apoptosis resembling Burkitt’s lymphoma. Some fell in between resembling “grey zone” lymphoma (Fig. 2A). These features were graded from grade 1 for “no tumor” to grade 6 for the high-grade Burkitt-like lymphoma. Grading of all the samples show that, in general, the tumors observed in *Eµ-myc Mtap^ +/−^* are of a higher grade than those in *Eµ-myc Mtap^+/+^* (Fig. 2C). The proliferation marker Ki67 was also examined and scored blindly, and it was found that there were increased numbers of strongly staining cells (up to almost 100%) in *Eµ-myc Mtap^lacZ/+^* animals (Fig. 2B–2C). Because loss of MTAP was associated with increased ODC activity in other settings, we stained thymus sections with an anti-body to mouse ODC. We observed both a higher percentage of cells expressing ODC and increased intensity of staining in the lymphomas from *Mtap^lacZ/+^* compared to *Mtap^+/+^* animals (Fig. 2B–2C). These findings show that the B-cell lymphomas in *Eµ-myc Mtap^lacZ/+^* animals tend to be of higher grade and have elevated ODC expression compared to *Eµ-myc Mtap^+/+^* animals.

### Mtap does not Affect the Developmental Stage of the Cell, Giving Rise to the Tumor

Because of both the earlier appearance and the increased grade of the tumor, our next question was whether *Mtap^lacZ/+^* altered the transformation stage of the lymphomas in *Eµ-myc* B cells. To address this question, we performed FACS analysis on tumor-infiltrated tissues including thymus, spleen, lymph node, and bone marrow. As shown in Table 3, we found that, with one exception (mouse 353), all of the lymphoma cells stained positive for CD19, CD45R/B220 and high AA4.1 (CD93) expression and negative for CD5 and CD3, indicating that they are early stage B-cells, either surface IgM^−^ or IgM^+^, in both *Mtap^+/+^* and *Mtap^lacZ/+^* mice. All AA4^+^ IgM^+^ cells were IgD^lo^ or IgD^−^, CD24^++^, CD21^−^, CD23^−^, in further agreement with their immature B cell stage. All IgM^−^ cells failed to show significant *TdT* mRNA levels, in contrast to the tight *TdT* (Terminal deoxynucleotidyl Transferase) expression by the pro B cells [34], and all expressed low levels of cytoplasmic IgM and high surface PNA expression, consistent with pre-B cell stage [41]. Low cytoplasmic IgM level excluded the possibility of IgM^−^ plasmacytoma. Taken together, our data show that the cell of origin of the lymphomas was most likely started from the pre-B stage of development in both *Mtap^+/+^* and *Mtap^−/−^* animals.

**Table 3:** FACS Analysis of *Em-myc Mtap^+/+^* and *Em-myc Mtap^lacZ/+^* mice.

Genotype (all *Em-myc*)	mouse	CD19	AA4.1	PNA	IgM	IgD	CD3	*TdT* (qPCR)	Cµ (qPCR)
*Mtap^+/+^*	370	+	+	++	−	−	−	−	+
*Mtap^+/+^*	322	+	+	++	+/−	−	−	−	+
*Mtap^+/+^*	329	+	+	++	++	+/−	−	nd	nd
*Mtap^+/+^*	331	+	+	++	++	−	−	nd	nd
*Mtap^+/+^*	336	+	+	++	++	−	−	nd	nd
*Mtap^+/+^*	353	−	−	−	−	nd	+	nd	nd
*Mtap^lacZ/+^*	309	+	+	++	−	−	−	−	+
*Mtap^lacZ/+^*	343	+	+	++	−	−	−	−	+
*Mtap^lacZ/+^*	369	+	+	++	−	−	−	−	+
*Mtap^lacZ/+^*	341	+	+	++	+/−	−	−	−	+
*Mtap^lacZ/+^*	320	+	+	++	++	+/−	−	nd	nd
*Mtap^lacZ/+^*	334	+	+	++	++	+/−	−	nd	nd

### Loss of Mtap Protein Expression in Lymphoma Cells

**Figure 3:**
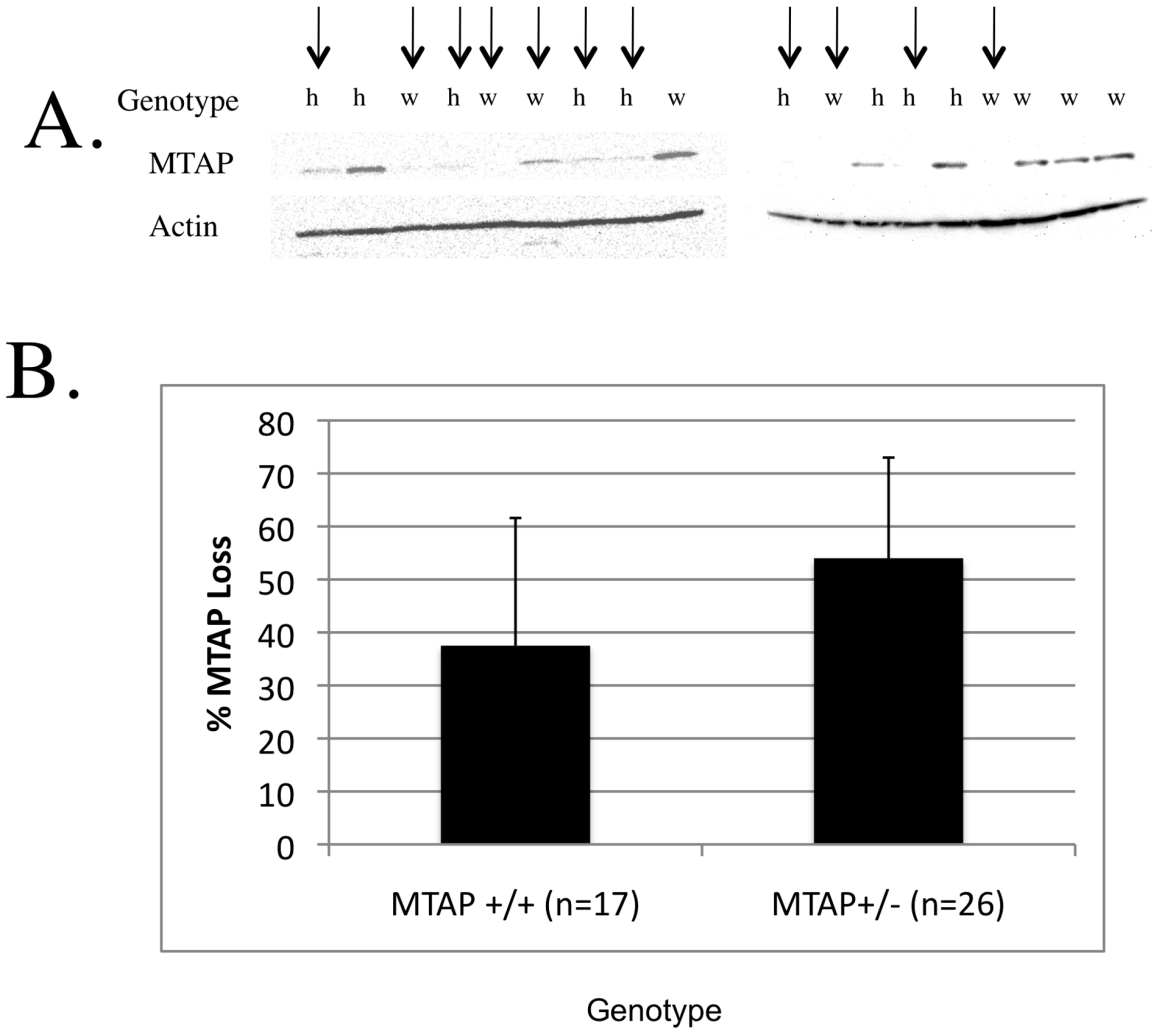
Loss of MTAP expression in lymphoma infiltrated tissue in *Eμ-myc Mtap^+/+^* and *Eμ-myc Mtap^lacZ/+^* mice. A. Representative Western blots showing MTAP protein in a variety of *Eµ-myc Mtap^lacZ/+^* (h, heterozygous) and *Mtap^+/+^* (w, wild type) animals. The arrows above the figure show the tumors that were scored as Mtap^−^. B. Bar Graph summarizing Western blot data for all 28 animals examined (P = ns). The average age of each of the animals making up each group is marked on the top of each column. Error bars show 95% confidence range.

We next examined Mtap expression in lymphoma-infiltrated tissue from 26 *Mtap^lacZ/+^* and 17 *Mtap^+/+^* animals by Western blot analysis (Fig. 3A). We found that 13/26 (50%) of the tumors from *Mtap^lacZ/+^* mice showed complete loss of MTAP protein compared to 5/17 (29%) of the tumors from *Mtap^+/+^* mice, but this difference was not statistically significant (P = 0.22, Fig. 3b). Given the large difference in tumor latency times between *Mtap^lacZ^* and *Mtap^+/+^*, these findings suggest that a conventional Knudson two-hit tumor suppressor model is not able to fully explain the differences in tumor formation kinetics and tumor severity between *Mtap^lacZ/+^* and *Mtap^+/+^* mice.

### Comparison of Gene Expression Profiles in Mtap^+/+^ and Mtap^lacZ/+^ Animals

**Figure 4:**
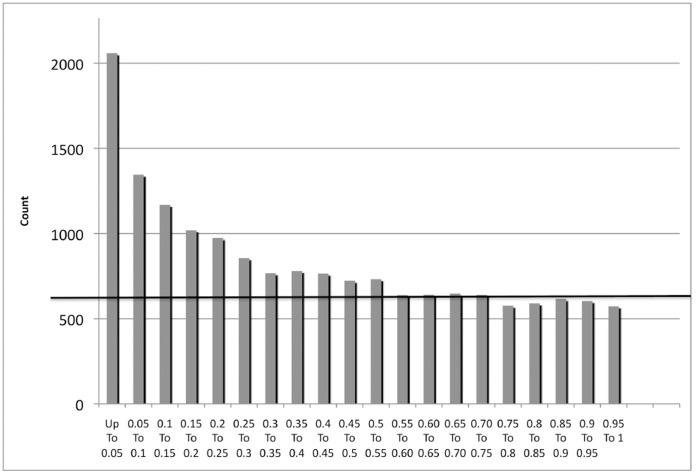
Histogram of P-values between *Mtap^+/+^* and *Mtap^lacZ/+^* livers. Line shows theoretical distribution of the null hypothesis (no differences in gene expression, P<0.0001).

Given the findings above, we hypothesized that mice heterozygous for *Mtap* might have phenotypes due to *Mtap* haploinsufficiency. To test this idea, we performed microarray expression analysis using Affymetrix chips on liver mRNA from a group of young, healthy, age and sex matched *Mtap^lacZ/+^* and *Mtap^+/+^* animals. Young mice were chosen as we anticipated that there gene expression profiles would have less overall variability due to the effects of aging and, therefore, would be more likely to observe statistically significant effects. The liver was chosen because of the livers central importance to amino acid metabolism. An examination of the distribution of P-values (Fig. 4) from the 16,717 probes that were expressed above background, clearly showed a significant enrichment in probes with P-values <0.05 (2,059 observed vs. 835 expected, P<0.0001). This finding shows that heterozygosity for a null allele of *Mtap* has a significant effect on the mRNA levels of a large number of genes.

To explore this further, we selected a group of 363 probes that exhibited at least a 50% change in mRNA levels with P<0.01 (FDR <0.29). Of these, 242 were up regulated and 121 were down regulated in *Mtap^lacZ/+^* vs. *Mtap^+/+^*. As expected, all four of the probes for Mtap were present in the down-regulated group. The remaining 359 probes mapped to 251 unique genes (see Table S1). We searched for functional enrichment of specific pathways of these genes using the Web Gestalt Gene Analysis Toolkit V2 [36]. Mapping our differentially expressed gene set against the biological function annotations in the Gene Ontology database, we found significant enrichment of genes involved rhythmic processes (i.e. circadian rhythm), anti-apoptotic genes, and genes involved in amino acid peptidyl modifications (Table S2). Another interesting group that came up as being enriched were genes involved in immature B-cell differentiation. Using the Kegg database as our functional sorter, we found that several probes mapped to signaling pathways including mTOR signaling, insulin signaling, and adipocytokine signaling, although these enrichments did not achieve statistical significance when correcting for multiple comparisons (Table S3).

We also subjected the same list of to analysis by the IPA software. The top five networks identified were: 1) Lipid Metabolism, Molecular Transport, Small Molecule Biochemistry (score 44); 2) Cancer, Endocrine System Disorders, Hematological Disease (score 31); 3) Cell Morphology, Cancer, Developmental Disorder (score 29) 4) Humoral Immune Response, Protein Synthesis, Hematological System Development and Function (score 25); and 5) Cell-To-Cell Signaling and Interaction, Skeletal and Muscular System Development and Function (score 25). A list of the cancer related genes identified by IPA is shown in Table S4.

The finding of a significant number of cancer related genes in the differentially regulated gene set is consistent with the idea that loss of a single *Mtap* allele may have protumorigenic affects.

We also examined transcripts of genes known to be involved in polyamine biosynthetic and degradation pathways (Table S5). We found that the transcripts for the polyamine catabolic enzyme spermidine/spermine N1-acetyl transferase 1 (*Sat1*) were increased in *Mtap^lacZ/+^* animals, while the biosynthetic gene spermidine synthase (*Srm1*) was down regulated.

## Discussion

Despite the knowledge that loss of MTAP expression is a relatively frequent event in a variety of different tumor types, the biological importance of MTAP loss in tumorigenesis has only recently started to be addressed. An important motivation for the studies described here was our earlier study in which we showed that mice heterozygous for *Mtap* died of T-cell lymphoma with a median life expectancy of 18 months [23]. Although this observation supported the idea that *Mtap* is a *bona fide* tumor suppressor gene, the long latency of this model makes it impractical for more extensive studies. An additional disadvantage of this model is that often *Mtap^lacZ/+^* mice would die of disease without exhibiting obvious external symptoms, making it difficult to get preserved tissue to study. To circumvent these problems, and to further establish that *Mtap* has tumor suppressor activity, we examined if a germline mutation in *Mtap* could cooperate and accelerate tumorigenesis in two other mouse tumor models, *Eµ-Myc* and *Pten^+/−^*. These models were chosen both because they have well defined tumor types and because they both have been used successfully to identify genetic interactions with other tumor suppressor genes such as p53, ARF, and CDN2K [42], [43].

Our data clearly show that heterozygosity for *Mtap* decreases tumor free survival in *Eµ-Myc* mice, with the median time for detectable tumor formation or death decreasing by 33%. For Pten^+/−^ mice, we did observe reduced survival, but did not observe a statistically significant increase in tumor formation the necropsied *Mtap^lacZ/+^ Pten^+/−^* animals. The reason for this apparent contradiction is that a larger percentage of the Mtap^lacZ/+^ animals died spontaneously and the samples were too badly decayed to be necropsied. Whether these animals died of tumors cannot be definitively determined. In retrospect, a proactive necropsy done at a particular time point probably would have been a superior strategy. On the other hand, *Eµ-myc Mtap^lacZ/+^* mice developed rapidly forming tumors that were easily detected by observing swollen lymph nodes in the neck of the effected mice. Histopathologic and FACS analysis of the lymphomas indicate that the cells of origin are pre-B and immature B cells, and that this cell type was the same for both *Mtap^+/+^* and *Mtap^lacZ/+^* animals. This finding indicates that *Mtap^lacZ/+^* can cooperate with *myc* in driving lymphoma formation and that *Mtap^lacZ/+^* does not alter the developmental stage of the cells giving rise to the lymphoma. However, we found that the tumors from *Mtap^lacZ/+^* animals were of a higher grade as judged both by cell morphology and staining for the proliferation marker Ki67. This, along with the earlier appearance of the tumors, suggests that loss of *Mtap* may cause increased tumor aggressiveness.

We also examined the frequency by which *Mtap* expression was lost in the lymphomas developed in *Eµ-myc Mtap* mice. We found that 5/17 tumors (29%) from *Mtap^+/+^* mice had lost Mtap expression compared to 13/26 (50%) from the *Mtap^lacZ/+^* animals. Although the frequency of Mtap- tumors appeared to increase in *Mtap^lacZ/+^* animals, this increase was not statistically significant and is unlikely explain the dramatic decrease in latency time observed in the *Mtap^lacZ/+^* animals. Rather, this data suggests that *Mtap* may be acting in a haploinsufficient manner. To develop evidence that germline heterozygosity for Mtap can have phenotypic consequences, we performed microarray experiments examining gene expression profiles in the livers of young age and sex matched *Mtap^+/+^* and *Mtap^lacZ/+^* animals. Based on the skewed distribution of P-values of the probes, we estimate that as many as 2048/16716 probes examined (14.4%) may be differentially expressed. Confining ourselves to probes that show at least a 50% difference in expression levels, we identified at least 363 probes representing 251 unique genes. These genes include many genes involved in pathways implicated in cancer development and progression. Because these experiments were done using RNA derived from liver, it is unclear if the genes and pathways identified as being affected by *Mtap* are directly relevant for the accelerated lymphoma development in these animals. Nonetheless, these experiments clearly show that loss of a single *Mtap* allele can have significant biological effects.

Previous studies have shown a relationship between loss of *Mtap* and an up-regulation of ODC, a key enzyme affecting polyamine metabolism [3], [20], [26]. In the studies described here, we found that the tumors in *Eµ-myc Mtap^lacZ/+^* mice tended to have higher levels of ODC expression than tumors found in *Mtap^+/+^* animals. In addition, we found Mtap-dependent differences in the liver mRNA levels of two polyamine metabolic genes (*Sat*1 and *Srm*1). Taken together, these observations provide additional support that Mtap-loss affects polyamine metabolism. A possible mechanism by which elevated ODC may contribute to lymphomagenesis may be via its influence on apoptosis. In hematopoietic cell lines, high levels of ODC have been shown to suppress apoptosis by reducing intracellular ROS species [44], [45]. However, it should be noted that loss of *Mtap* might also promote lymphomagenesis by other means as well. In unpublished studies, our lab has found that expression of *Mtap* in an *Mtap* deleted osteosarcoma cell line can suppress several tumor related phenotypes without any effect on ODC levels (W.K., unpublished data). Thus, it seems possible that there may be multiple mechanisms by which *Mtap*-loss promotes tumor formation.

In summary, we have shown here, for the first time, that germline mutations *Mtap* can cooperate genetically with at least two other cancer causing mutations, *Eµ-myc* and *Pten^+/−^*, to reduce survival and, in the case of E*µ*-myc, accelerate tumorigenesis. This acceleration does not appear to require the loss of the wild-type *Mtap* allele, suggesting that loss of a single copy of *Mtap* may have protumorigenic affects. Consistent with this view is the observation that heterozygosity for *Mtap* results in large alterations in the liver gene expression profile. Our findings support the view that *Mtap*-loss is of biological importance in tumorigenesis.

## Supporting Information

Table S1 **Mtap differentially expressed genes.**
(XLSX)

Table S2 **Gene Ontology Pathways affected by Mtap.**
(XLSX)

Table S3 **Kegg Pathways affected by Mtap.**
(XLSX)

Table S4 **Cancer genes identified by IPA analysis.**
(XLSX)

Table S5 **Analysis of Polyamine Pathway genes.**
(XLSB)
